# Primary care nurses’ preparedness for COVID-19 in the Western Cape province, South Africa

**DOI:** 10.4102/phcfm.v13i1.2879

**Published:** 2021-05-28

**Authors:** Talitha Crowley, Danine Kitshoff, Frances de Lange-Cloete, Justine Baron, Santel de Lange, Cornelle Young, Tonya Esterhuizen, Ian Couper

**Affiliations:** 1 Department of Nursing and Midwifery, Faculty of Medicine and Health Sciences, Stellenbosch University, Cape Town, South Africa; 2 Department of Global Health, Faculty of Medicine and Health Sciences, Stellenbosch University, Cape Town, South Africa; 3 Division of Epidemiology and Biostatistics, Department of Global Health, Faculty of Medicine and Health Sciences, Stellenbosch University, Cape Town, South Africa; 4 Ukwanda Centre for Rural Health, Department of Global Health, Faculty of Medicine and Health Sciences, Stellenbosch University, Cape Town, South Africa

**Keywords:** COVID-19, primary care, nurses, preparedness, Western Cape

## Abstract

**Introduction:**

The novel coronavirus 2019 or COVID-19 pandemic has brought about a global public health crisis. Primary care (PC) nurses render first line care, or refer for more specialised services.

**Aim:**

To investigate the preparedness of PC nurses for COVID-19 in the Western Cape.

**Setting:**

The Western Cape province of South Africa.

**Methods:**

We administered an online survey, with closed and open-ended questions, to 83 Stellenbosch University postgraduate PC nursing students and alumni working in the Western Cape, between 03 July and 01 September 2020.

**Results:**

The results indicated that 43.3% of participants were confident about the infection, prevention, and control (IPC) training they received and 56.7% felt prepared to provide direct care to suspected cases of COVID-19. Primary care nurses were more comfortable to triage (78.3%) than to manage persons with COVID-19 (42.2%), indicating that they may not be functioning to the full capacity of their education and training. Adequate infrastructure was reported by less than a third of the participants (30.1%) and 59.1% reported that personal protective equipment (PPE) was always available. Primary care nurses needed support in coping with stress (57.8%) although few (14.5%) reported access to mental health services.

**Conclusion:**

Primary care nurses were not prepared optimally for the COVID-19 pandemic. Challenges included adequate training, infrastructure, the availability of personal protective equipment, COVID-19 testing of health care workers and management support. Primary care nurses need comprehensive support to manage stress and anxiety.

## Introduction

The novel coronavirus (Severe Acute Respiratory Syndrome [SARS]-Cov-2) or coronavirus disease 2019 (COVID-19) pandemic has brought about a global public health crisis.^1^ Primary care (PC) is the first point of entry into the healthcare system for many people and it is here where PC workers play a pivotal role in the response to and management of infectious diseases to ensure early identification and prevent the spread thereof. Primary care nurses are the leading frontline healthcare providers in community settings and are central to ensuring universal health access and achieving the sustainable developmental goals.^2^

The South African healthcare system offers a range of preventative and curative services at PC level, including chronic care to address the quadruple burden of disease.^3^ Not only have PC workers continued to provide these services during the pandemic, they have also needed to conduct COVID-19 screening and testing. Managing these additional workloads because of an increased number of people accessing the services and managing their own infection risk may be very stressful.^1,4^

To minimise the spread of COVID-19, routine droplet and contact precautions, environmental hygiene and overall infection prevention and control precautions are needed. Recommendations on workplace preparedness state that healthcare workers who are exposed to possible or known COVID-19 cases are classified as having a high exposure risk.^5^ Healthcare workers must assess their own risk, self-monitor and report if they experience symptoms. Those with unacceptably high risk because of other conditions should be redeployed. Strict adherence to guidelines and precautions is central to the protection of healthcare workers.^1^

In April 2020, the Infectious Diseases Society of South Africa published a COVID-19 Primary Care Preparedness Guide.^6^ The guide specifies the requisite equipment and consumables, required training, triage, management and referral procedures needed for the COVID-19 response.^6^ Despite these guidelines, PC workers may not have access to the required facilities, equipment, consumables and training. Even internationally in high-income countries, there is limited availability of personal protective equipment (PPE) and respiratory isolation rooms to adequately evaluate patients.^1^ To ensure nurses’ protection during the COVID-19 pandemic, the following is advised: intense education and training; reasonable shift schedules; making full use of existing infection, prevention and control (IPC) systems; providing psychological counselling; and avoiding unnecessary contact.^7^

It is likely that nursing curricula do not cover pandemic preparedness adequately, as this pandemic is the first experienced on such a scale, the previous ones (e.g. Middle East Respiratory Syndrome [MERS] and SARS) not reaching such global proportions. Although general disaster management is addressed in all postgraduate diploma in nursing programmes at Stellenbosch University, existing general guidelines, and those discussed in curricula for the safety of healthcare workers during a pandemic, are not comprehensive and precise.^4^ Nurses thus need to attend additional training to provide COVID-19 services. There is still a lack of definitive evidence supporting which knowledge and skills are required to ensure that nurses perform competently during pandemics.^8^ The competencies may also be different depending on the setting and the services provided.

Research on the experiences of frontline healthcare workers, such as PC nurses, in the early phases of a pandemic is crucial to inform strategies to ensure better support and future planning. For example, during the early phases of the 2009 H1N1 pandemic, a quantitative study in England found that some healthcare workers had negative attitudes towards taking antivirals and receiving influenza vaccinations.^9^ The same study also found that the main source of information about infection control and risk was local guidance. In addition to work stressors, healthcare workers may be concerned about the risk of infecting family members, necessitating measures to prevent transmission when arriving at home or for self-quarantine when they are persons under investigation (PUI).^1,9^ Other concerns include childcare responsibilities and dependents requiring their care and support.^10^ It is therefore important to determine the COVID-19 workplace-based preparedness as well as psychological preparedness of PC workers.

The impact of COVID-19 on the Nursing and Midwifery workforce study (ICON) in the United Kingdom found that of 2600 nurses who responded, 92% were worried about risks to family members and 74% felt their own health was at risk.^11^ More than half (52%) of the participants did not have sufficient confidence or training about COVID-19 infection and one-third (33%) reported severe or extremely severe anxiety, depression or stress. Almost two-thirds of participants (62%) had inadequate or no redeployment training.^11^ These findings indicate a need for context-specific research to investigate the preparedness of PC nurses for COVID-19.

The aim of this study was to investigate the preparedness of PC nurses for COVID-19 in the Western Cape province, South Africa.

## Methodology

### Design

A quantitative descriptive research design was used. We conducted an online survey using RedCap that was sent to Stellenbosch University’s postgraduate diploma in PC nursing students and alumni.

### Setting

The Department of Nursing and Midwifery at Stellenbosch University is responsible for the postgraduate training of PC nurses. The students come from various urban and rural districts in the Western Cape. These students and alumni were the most accessible population to perform a rapid assessment of the current situation.

### Instrument

The questionnaire was developed by the research team and was based on the literature, ‘The impact of COVID-19 on the nursing and midwifery workforce study (ICON)’, questions for this specific category of healthcare worker^11^ and the team’s own experience. The questionnaire consisted of 48 questions, both closed- and open-ended, in seven sections. Section A consisted of demographic information such as age, sex, qualification, position and district where working. Section B required information about COVID-19 training and attitudes. Section C required information about access to guidelines. Section D related to facilities and equipment. Section E enquired about services reorganisation. Section F consisted of information and training needs. Section G enquired about personal and self-care needs. Open-ended questions allowed the participants to explain their answers to certain responses and provided an opportunity to elicit complementary information. For the purpose of this article, we focused on the sections related to the preparedness of PC nurses. The questionnaire is available on request from the authors.

Four experts, including health service managers and academics involved in planning for the COVID-19 pandemic in the Western Cape, reviewed the questionnaire to establish face validity and assessed the alignment between the research objectives and the questions. Reliability analysis of the two Likert-scale questions related to confidence in training and preparedness indicated a Cronbach’s alpha of 0.7. The three Likert-scale questions related to stress and worry about COVID-19 had a Cronbach’s alpha of 0.75, indicating an acceptable reliability.

### Population and sample selection

**FIGURE 1:**
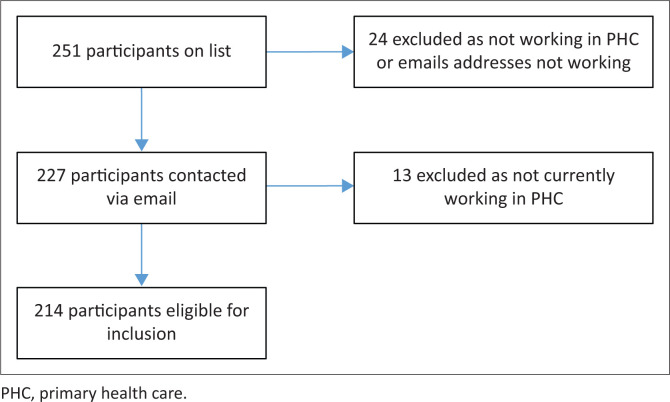
Sample selection.

Current PC nursing students (year 2020) and students from the years 2017–2019 were included (*N* = 251) in the study. Figure 1 depicts the study sample.

### Pilot test

We conducted a pilot test of the questionnaire on 12 conveniently selected PC nurses of the 2016 student cohort. The purpose of the pilot test was to determine if the questions were clear. We contacted the participants after they completed the questionnaire online via WhatsApp to enquire if they experienced any difficulty completing the questionnaire and to obtain feedback. Minor changes were made following the pilot test and the responses from the pilot test were not included in the main study.

### Data collection

Once institutional permission was obtained, an email with a link to the online consent form and questionnaire was sent to all the eligible students and alumni. Follow-up emails were sent via RedCap to remind participants to complete the questionnaires. Participants who did not respond after several reminders received a courtesy WhatsApp message to ask them if they were aware of the survey, in an attempt to increase the response rate and representativeness of the study results. This did not compromise confidentiality, as RedCap only indicated which respondents did not respond; the participants’ identities are not linked to their responses. The electronic link was open from 03 July 2020 to 01 September 2020, although the majority of the participants completed the questionnaires between 03 July 2020 and 31 July 2020.

Participants who completed the questionnaire were entered into a lucky draw to win a gift voucher to the value of R1000.00. This incentive was introduced in an attempt to increase the participant response rate.

### Data analysis

Data were analysed descriptively and summarised in frequency tables or graphs. Inferential statistics included differences between urban and rural districts and participant responses, using the chi-square test. Content analysis^12^ was used to analyse the open-ended questions. Themes were identified and quantified.

### Ethical considerations

We considered the ethics and practicalities of duty of care during pandemics.^13^ Duty of care also drove this research as we aimed to determine the preparedness of PC nurses for the COVID-19 pandemic in order to make recommendations on how to improve support.

Ethical approval was obtained from the Health Research Ethics Committee at Stellenbosch University (clearance no. N20/04/015_COVID-19) and institutional permission was obtained to access the students’ contact details. This research was of minimal risk as all data collection occurred online and did not compromise the health of any participants or researchers. We specifically kept the questionnaire short so that it did not require too much time from the participants who may be providing essential services. Participants agreed to participate after reading an online participant information leaflet.

## Results

### Demographic information

The final sample included 83 participants (83/214, 38.7%). The mean age of the participants was 37.8 years (standard deviation [s.d.] 7.3 years), with a mean number of years working in Primary Health Care (PHC) of 5.4 (s.d. 4.8). Of the sample, 85.5% (71) were female participants and the majority (69, 83.1%) indicated that their highest qualification was a postgraduate diploma in nursing. Table 1 indicates the category and area of work. Most of the participants (49, 59%) worked in urban districts. The majority worked in public health facilities (63, 75.9%).

**TABLE 1:** Category and area of work.

Variable	Frequency	Percentage
**Category**†		
Clinical nurse practitioner	40	48.2
Professional nurse	40	48.2
Facility manager	1	1.2
Other (e.g. HAST coordinator, midwife, operational manager, senior professional nurse, student clinical nurse practitioner)	9	10.8
**Facility**†		
Public health clinic	32	38.6
Public community health centre	26	31.3
Public mobile clinic	5	6.0
Private clinic	7	8.4
Other: correctional services, home-based care, non-governmental organisations, military, training	16	19.3
**District (*n* = 83)**		
Urban: Metro – City of Cape Town	29	34.9
Urban: Metro – Department of Health	20	24.1
Rural: Cape Winelands	13	15.7
Rural: Eden	6	7.2
Rural: Overberg	4	4.8
Rural: West Coast	4	4.8
Rural: Karoo	4	4.8
Missing	3	3.6

HAST, HIV/AIDS, sexually transmitted infections and tuberculosis

† , Multiple response options, so frequencies do not add up to 100%.

### Training and attitudes

In response to questions on training to triage and manage COVID-19, some participants indicated that they received no training (*n* = 10, 12.0%). The most common training method was an instructional video (*n* = 28, 33.7%) (see Table 2). Less than half of the participants (*n* = 36, 43.3%) felt confident or very confident about the IPC training received in relation to COVID-19 and just more than half (*n* = 47, 56.7%) felt somewhat or very prepared to provide care. More participants indicated having the necessary expertise to triage patients with COVID-19 (*n* = 65, 78.3%) than to manage persons with COVID-19 (*n* = 35, 42.2%).

**TABLE 2:** Training and confidence to triage and manage coronavirus disease 2019.

Variable	Frequency	Percentage
**Type of training**†		
Formal instructional video	28	33.7
Written instruction	27	32.5
Training on what PPE to wear for different activities	27	32.5
Departmental guidance	26	31.3
Formal classroom training (online and/or face-to-face)	24	28.9
Workplace-based follow-up and support	22	26.5
Formal fit testing for masks	14	16.9
Simulation training (e.g. practice with real equipment)	13	15.7
No training	10	12.0
**How confident do you feel in the infection prevention and control training that has been provided to you in relation to COVID-19? (*n* = 83)**		
Not received any training	10	12.0
Not confident at all	5	6.0
Not very confident	17	20.5
Neither not confident nor confident	14	16.9
Confident	31	37.3
Very confident	5	6.0
Missing	1	1.2
**How prepared do you feel to provide direct care to suspected cases of COVID-19? (*n* = 83)**		
Completely unprepared	7	8.4
Somewhat unprepared	9	10.8
Neither unprepared or prepared	19	22.9
Somewhat prepared	33	39.8
Very prepared	14	16.9
Missing	1	1.2
**Do you feel that you have sufficient expertise to triage or screen patients with possible COVID-19? (*n* = 83)**		
Yes	65	78.3
No	17	20.5
Missing	1	1.2
**Do you feel that you have sufficient expertise to manage patients with COVID-19? (*n* = 83)**		
Yes	35	42.2
No	47	56.6
Missing	1	1.2
**How comfortable are you with providing healthcare to patients with possible COVID-19? (*n* = 83)**		
I am not willing to provide care	3	3.6
Uncomfortable	18	21.7
Somewhat comfortable	32	38.6
Comfortable	23	27.7
Very comfortable	6	7.2
Missing	1	1.2
**With regard to your answer to the previous question, please specify why**‡		
I think it is important to provide care to COVID-19 patients	49	59.0
I am worried that I may transmit the virus to my family members	41	49.4
Fear of possible exposure	34	41.0
I do not have sufficient training	18	21.7
I have underlying health conditions putting me at risk of developing complications if I become infected with COVID-19	13	15.7
I am currently or have been infected with COVID-19	9	10.8
I have a high risk of acquiring COVID-19	8	9.6
I am afraid of the stigma of COVID-19 in my community	6	7.2
Unwillingness to be exposed because of lack of vaccine or treatment	4	4.8
Other: ‘I was infected with COVID-19 that led me infecting my family.’	1	1.2

PPE, personal protective equipment; COVID-19, coronavirus disease 2019.

† , Multiple response options, so frequencies do not add up to 100%;

‡ , Multiple response options.

Very few participants (*n* = 3, 3.6%) indicated that they were not willing to provide care to persons with COVID-19, whilst 34.9% (*n* = 29) were comfortable or very comfortable to provide care. More than half of the participants reported that it is important to provide care to persons with COVID-19 (*n* = 49, 59.0%). Discomfort mostly related to being worried about family members (*n* = 41, 49.4%), fear of exposure (*n* = 34, 41.0%) and having underlying health conditions (*n* = 13, 15.7%).

We used two open-ended questions to ask participants about their information and training needs. Table 3 summarises the themes identified.

**TABLE 3:** Information and training needs.

Themes	Quotation	Frequency†	Percentage
**Information needs (*n* = 49)**			
In-service training	‘… [M]ore information, for example, the correct PPE for staff members because we are not doing the same thing and now if you are infected you are being blamed for carelessness’.	30	61.2
Communication	‘… Internal communication is vital I think because certain staff members tend to slack down when it comes to distancing in the workplace and taking breaks by overcrowding the tea rooms …’	22	44.9
Vaccines, immunity, re-infection	‘Constant update on the progress made in the search for a vaccine’.	6	12.2
Staff wellness	‘Support services being made available to the healthcare personnel. More emotional support, because family duties’.	3	6.1
Health promotion	‘I think there is a lack of posters and billboards in our communities highlighting COVID-19’.	1	2.0
**Training needs (*n* = 47)**			
Skills development	‘Screening and testing of patients. Referrals when necessary and management if possible’.	28	59.6
In-service training	‘… Emphasis on the pandemic within the workplace should continue to allow for staff members also to maintain adherence in risk reduction …’	8	17.0
Communication	‘Stop giving new SOPs every week while the old one isn’t even implemented’.	3	6.4
Health promotion	‘How to keep my patients and myself safe from contracting COVID-19. Education that I can offer to my family/subordinates/community and to the congregation at large’.	1	2.1

PPE, personal protective equipment; SOP, standard operating procedures; COVID-19 coronavirus disease 2019.

† , Frequencies and percentages were calculated out of the number of participants who responded and represent the frequency of the themes in the participant narratives.

### Guidelines

The majority of participants had access to guidelines on triaging and managing COVID-19 (*n* = 71, 5.5%) and access to the use of PPE (*n* = 72, 86.7%). Guidelines that were the most often used were the Practical Approach to Care Kit (*n* = 56, 67.5%) and the Western Cape Department of Health guidelines (*n* = 44, 53%). A large majority of participants reported that they followed the guidelines (*n* = 76, 91.6%) and 84.3% (*n* = 70) found the guidelines helpful.

In the open-ended question, participants reported reasons for not finding the guidelines helpful, such as lack of clarity about what PPE to wear in certain situations, especially in emergency units because of uncertainty of whether a patient might have COVID-19, continuous changes in the guidelines or a lack of training on guidelines. One participant commented:

‘The guidelines don’t take appropriate infrastructure of facility in mind … still don’t take health care practitioners’ expertise or the amount of patients in mind for effective triage and management.’ (Male, 31 years, community health centre)

### Personal protective equipment and infrastructure

Less than one-third of the participants (25, 30.1%) indicated that there was adequate infrastructure in their facility to triage and manage COVID-19 cases and almost two-thirds of the participants (*n* = 49, 59.1%) agreed or strongly agreed that PPE was always available.

In the open-ended question, participants indicated several infrastructure and equipment needs including full PPE packs for testing, dedicated space for COVID-19 triage and testing, isolation rooms, patient flow challenges such as the inability to effectively separate patients because of load and small spaces with a lack of ventilation. In most facilities, COVID-19 screening and triaging was performed outside, for example, in gazebos or tents. These spaces were not always suitable because of windy and rainy weather conditions. Many participants reported a lack of equipment that was fully functional and sharing equipment between COVID-19 and non-COVID-19 patients.

Mobile clinics experienced particular challenges as shown in the below quote:

‘At mobile clinic, screening is outside. Privacy problem. In rain, sick patient cared for in mobile clinic. Little space, distancing impossible. In level 5 lockdown we were told by management, no need for masks, only distancing and handwashing/sanitizing. Received first two N95 masks in late April and forehead thermometer end of June.’ (Female, 53 years, mobile clinic)

One participant commented that theft and the incorrect use of PPE had led to a lack of PPE availability:

‘This has resulted in all PPE being locked away and only available when signing for it. Many of our staff have a sense of panic and paranoia and tend to use the incorrect PPE; this has resulted in shortages that could have been avoided.’ (Female, 51 years, district hospital)

### Personal and self-care needs

More than half of the participants indicated that they needed support for coping with stress (*n* = 48, 57.8%), whilst 41% (*n* = 34) frequently or very frequently experienced feelings of distress related to COVID-19; however, only 33.7% (*n* = 28) were concerned about their self-care needs (see Table 4). One participant’s comment may explain why so few were concerned about their self-care needs:

‘Due to the nature of my work I have to put the patient first; therefore, my personal health always comes last.’ (Male, 43 years, correctional services)

**TABLE 4:** Personal and self-care needs.

Variable	Frequency	Percentage
**Do you have any caring responsibilities (e.g. child or adult family member) (*n* = 83)**		
Yes – sole carer	24	28.9
Yes, but not sole carer	38	45.8
No	19	22.9
Missing	2	2.4
**I need support with the following responsibilities**†		
Coping with stress (psychological needs)	48	57.8
Home schooling	24	28.9
Childcare	22	26.5
Taking care of family members	22	26.5
None	13	15.7
Other: ‘Husband lost work’	1	1.2
**What support measures have been put in place by your manager or employer since the COVID-19 outbreak?**†		
Daily symptom screening	75	90.4
Implementation of safe work practices	39	47.0
COVID-19 testing	28	33.7
Employee wellness	22	26.5
Guidelines for coping and managing burnout	13	15.7
Access to mental healthcare	12	14.5
Buddy support	8	9.6
None	4	4.8
Other: ‘Debriefing sessions were organized in groups of 5’	1	1.2
**During the past 7 days, how often did you experience feelings of distress with respect to COVID-19? (*n* = 83)**		
Not at all	6	7.2
A little bit of the time	12	14.5
Sometimes	30	36.1
Frequently	19	22.9
Very frequently	15	18.1
Missing	1	1.2
**I feel that my personal health is at risk during the COVID-19 outbreak because of my clinical role (*n* = 83)**		
Strongly agree	33	39.8
Agree	36	43.4
Neither agree nor disagree	9	10.8
Disagree	2	2.4
Strongly disagree	2	2.4
Missing	1	1.2
**How worried are you about the potential personal risks to become infected with COVID-19? (*n* = 83)**		
Extremely worried	39	47.0
Somewhat worried	30	36.1
Neither worried nor not worried	7	8.4
Mostly not worried	5	6.0
Not worried at all	1	1.2
Missing	1	1.2
**How worried are you about the potential risks to your family, loved ones or others because of your clinical role in the COVID-19 outbreak? (*n* = 83)**		
Extremely worried	62	74.7
Somewhat worried	13	15.7
Neither worried nor not worried	2	2.4
Mostly not worried	4	4.8
Not worried at all	1	1.2
Missing	1	1.2
**I am concerned about my own self-care needs (*n* = 83)**		
Yes	28	33.7
No	53	63.9
Missing	2	2.4

COVID-19, coronavirus disease 2019.

† , Multiple response options, so frequencies do not add up to 100%.

Themes related to self-care and mental health needs were identified in the open-ended questions. These included fear because of comorbidities such as diabetes and hypertension; uncertainty about PPE requirements; lack of ability to take care of personal health (exercise, rest, etc.) because of other responsibilities; fear of infecting a partner or family members (social life changes); difficulty isolating because of having children at home or not knowing what to do during isolation; and a lack of employer support, which are evident in the following quotes:

‘I have 2 kids, I am a single parent only stay with them; [*the*] first born is 16 years and [*the*] last born is 3 years and I don’t have a carer to look after them. I work Monday to Friday; no time for home schooling, I am working with high-risk clients.’ (Female, 30 years, non-governmental organisation)

‘When staff tested positive, they must hide the status, no support from company when one tested positive, no deep cleaning at all.’ (Female, 36 years, community health centre)

Participants made suggestions for improving their work life and these are depicted in Table 5.

**TABLE 5:** Suggestions for improving work life.

Themes (*n* = 72)	Example quote	Frequency†	Percentage
Effective leadership, management and support	‘The main thing that is lacking from our employer is enough support instead, we are issued with circulars that are read to us. Many of our staff that have tested COVID-positive are people that declared that they have an underlying medical condition but the circular says they are not high risk so they ended up working in COVID area and some of them were even admitted to hospital. And that has left us feeling very worried and concerned but the employer is only concerned about what to be done for patients when we have staff shortage due to quarantine of the staff’.	44	61.1
Staff wellness	‘I feel that government should value nurses, doctors and all health care professionals by [*implementing*] wellness programmes. A lot of my colleagues are stressed and not coping due to their own risk profile. I also feel that government should not withhold financial incentives like many other countries did and give us the increase in our annual salary as per wage agreement’.	37	51.4
Human resources, infrastructure and equipment	‘Attempt to increase the infrastructure. Increase staff for just screening and testing of patients. Provide more PPE’.	33	45.8
In-service training	‘Provision of training and enabling environment for nurses to perform their duties without fear’. ‘I want the training required to test patients at the facility I work at, instead of having to refer them to a primary health care facility and not only burdening the public health system but also putting employees at risk’.	10	13.8

PPE, personal protective equipment; COVID, coronavirus disease.

† , Frequencies and percentages were calculated out of the number of participants who responded and represent the frequency of the themes in the participant narratives.

Inferential statistics did not indicate any significant differences between rural and urban areas and the responses provided by the participants.

## Discussion

We set out to determine the preparedness of PC nurses for the COVID-19 pandemic in the Western Cape province. Only 56.7% of the participants indicated that they were partly or fully prepared to provide care to patients with COVID-19. Primary care nurses were more confident to screen possible PUI than to manage individuals with COVID-19. In the United Kingdom, 52.0% of nurses reported confidence in their training about COVID-19.^11^ In Iran, 56.5% of nurses reported good knowledge^14^ compared to 71.9% of PC nurses in Australia who believed that they had sufficient knowledge of COVID-19.^15^

The COVID-19 pandemic necessitated the training of PC workers, specifically nurses, to respond to patient care needs.^4,16,17^ This should ideally also include simulation training to facilitate critical thinking skills unique to pandemics.^18^ However, such training may be challenging because of rapid curriculum development and various social distancing restrictions on teaching and learning as well as staff being needed in the healthcare facilities and thus not able to attend training sessions. In line with this, the most often reported training platform was an instructional video, followed by written instruction. However, these methods were not sufficient, as indicated by the percentage of participants that either did not receive training or did not feel confident about the IPC training received (55.4%). Qualitative data indicated the need for further skills development and in-service training. Additional training will assist to ensure that PC nurses function to the full scope of their education and training, as research shows that they may provide equal or better care than doctors.^19^

Guidelines appeared to be available and accessible to most participants. This is contrary to an Australian study where only 47.3% of participants reported access to COVID-19-specific guidelines.^15^ Although COVID-19 guidelines were available, the application of the guidelines was problematic, particularly in certain contexts such as small clinics and mobile clinics with poor infrastructure. Poor leadership from management hindered guideline implementation. Guidelines changed during the course of the pandemic, which were problematic for some participants. It is recommended that guidelines should be adapted based on the pandemic stage or interval.^20^ This necessitates clear communication, which was highlighted in the qualitative data.

Less than one-third of participants reported adequate infrastructure, indicating that PC facilities are not tailored to manage patients during pandemics. Personal protective equipment were not always available in facilities. Similarly, in Australia and the United Kingdom, PC workers reported insufficient PPE.^15,21^ This highlights the need to strengthen primary healthcare systems globally^17^ and to employ strategies to better conserve and manage PPE.^22^

Congruent with other studies,^1,11,15,23^ 59% of PC nurses in the Western Cape considered providing COVID-19 services and care to be important. However, they were worried about the possible risk to themselves and their families. Only 34.9% felt comfortable or very comfortable to provide care to patients with COVID-19, compared to 59% of PC nurses in an Australian study.^15^ This indicates that concerns about family risk and mitigation strategies need to be addressed.^1^

Participants reported support needs such as childcare, home schooling and taking care of family members. Similar carer responsibilities were reported amongst Australian PC nurses.^15^ Almost 60% of participants needed support with coping and stress management, yet only 14.5% reported access to mental healthcare at their workplaces. Other studies also found that comprehensive assessment, support and management of anxiety during a pandemic are needed.^23,24^ A lack of support from management was identified in this study. In Australia, only 54.8% of PC nurses reported feeling supported by management.^15^ In our study, only 33.7% of the participants reported having access to COVID-19 testing at their workplace. This is concerning as clear strategies should be in place to manage exposed and infected PC workers.^25^

The study participants indicated that effective management and staff wellness were the most pertinent needs. A study in the United States of America found that healthcare workers requested to be heard, protected, prepared, supported and cared for during a pandemic.^24^ Investment in leadership and support for PC nurses are therefore essential.^17^

## Strengths and limitations

The limitations of the study include its cross-sectional nature, the relatively low response rate, the focus on the public health system and the dominance of respondents from urban areas. Furthermore, the fact that the questionnaire was administered online, and that the participants were all from the same university, could have introduced sampling or response bias. The inclusion of other universities within the Western Cape may have provided a more representative sample, but may have also delayed data collection during the peak of the pandemic. The findings, however, resonate with global literature. Further, the qualitative participant responses provide rich contextual information that increases the transferability of the findings.

## Conclusion

Primary care nurses remain central to ensure ‘health for all’ and continuous quality healthcare. The findings of this study indicate that PC nurses were not optimally prepared for the COVID-19 pandemic. Challenges included adequate training, infrastructure, the availability of PPE, COVID-19 testing of healthcare workers and management support. Personal care nurses need comprehensive support to manage stress and anxiety. The findings of this study may be used to inform nursing curricula and future PC preparedness interventions.
